# Desmosine: The Rationale for Its Use as a Biomarker of Therapeutic Efficacy in the Treatment of Pulmonary Emphysema

**DOI:** 10.3390/diagnostics15050578

**Published:** 2025-02-27

**Authors:** Jerome Cantor

**Affiliations:** School of Pharmacy and Allied Health Sciences, St John’s University, 8000 Utopia Parkway, Queens, NY 11439, USA; cantorj@stjohns.edu

**Keywords:** desmosine, pulmonary emphysema, elastin, elastic fibers, hyaluronan

## Abstract

Desmosine and isodesmosine (DID) are elastin-specific crosslinking amino acids that play a critical role in maintaining the structural integrity of elastic fibers, and their levels in body fluids may serve as biomarkers for alveolar wall injury. To support this concept, we present studies demonstrating the use of DID to detect elastic fiber damage that reflects distention and the rupture of airspaces. The emergence of airspace enlargement may be modeled by a percolation network describing the effect of changing proportions of intact and weak elastic fibers on the transmission of mechanical forces in the lung. Following the unraveling and fragmentation of weakened elastic fibers, the release of DID may correlate with an increasing alveolar diameter and provide an endpoint for clinical trials of novel agents designed to treat pulmonary emphysema. The limitations of the DID measurements related to specificity and reproducibility are also addressed, particularly regarding sample source and analytical techniques. Standardizing protocols to isolate and quantify DID may increase the use of this biomarker for the early detection of alveolar wall injury, which permits timely therapeutic intervention.

## 1. Introduction

**Figure 1:**
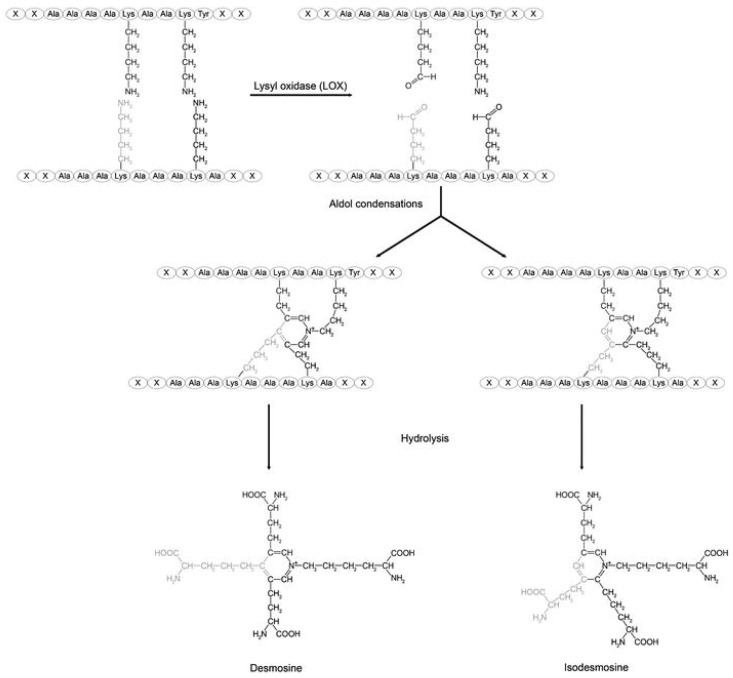
Desmosine and Isodesmosine are crosslinks formed by the condensation of four lysine residues on adjacent elastin peptides. The only difference between them is the positioning of a lysine side chain on the central pyridinium ring. Reprinted with permission of Creative Commons (https://creativecommons.org/licenses/by-sa/4.0/, accessed on 2 October 2024).

Desmosine and isodesmosine (DID) are elastin-specific crosslinking amino acids that play a critical role in alveolar wall mechanics (Figure 1) [1]. The ability of DID to form covalent bonds between elastin peptides contributes to the structural integrity and distensibility of elastic fibers. Understanding the relationship between DID and pulmonary disease is essential for determining its usefulness as a biomarker [2,3,4]. Increased DID levels in body fluids can reflect alveolar wall injury, making these crosslinks a potentially useful biomarker for detecting pulmonary emphysema and other diseases involving changes in lung mechanics. Studies have shown that elevated DID levels correlate with elastin degradation in patients with chronic obstructive pulmonary disease (COPD), a disease complex including chronic bronchitis and pulmonary emphysema [5,6,7,8]. This finding is supported by the measurement of DID in animal models that mimic the alveolar wall changes associated with COPD [9,10].

Based on these studies, the measurement of DID levels may aid in the clinical diagnosis of emphysema. While imaging techniques and pulmonary function tests provide valuable information regarding structural changes in lung tissue and airflow obstruction, they may not capture the biological processes underlying tissue damage [2,8]. Incorporating DID measurements into the diagnostic protocol may enhance accuracy by providing insight into the ongoing elastin breakdown. Elevated crosslink levels can signal significant emphysematous changes even when imaging appears normal or offers ambiguous results [5].

In patients diagnosed with emphysema, the regular monitoring of DID levels can serve as a biomarker for disease progression. Fluctuations in the DID concentration over time can reflect changes in the underlying disease process in ways that imaging alone may not capture. Consequently, the biomarker can be particularly beneficial for tracking the efficacy of therapeutic interventions and adjusting management plans accordingly. In cases where DID levels are significantly elevated, clinicians may prioritize treatments to address inflammation and tissue repair, such as inhaled corticosteroids or novel biologic therapies [6,7]. Conversely, stable DID levels may suggest the disease is under control, allowing for a more conservative management approach [8].

Furthermore, it may be possible to establish a relationship between DID levels and specific emphysema phenotypes [8]. Such studies could determine whether DID can stratify patients more effectively based on their disease characteristics, potentially leading to personalized treatment pathways. Additionally, exploring the relationship between DID and other lung injury biomarkers may yield a broader understanding of the biological processes involved in pulmonary emphysema.

### 1.1. Modeling the Role of Elastic Fiber Injury in Pulmonary Emphysema

In the early stages of pulmonary emphysema, localized damage occurs to the elastic fiber network of the lungs, resulting in only minor changes to the lung structure. However, as this damage progresses, it disrupts the normal distribution of mechanical forces, leading to the rupture of alveolar walls [11,12]. This process significantly reduces the lung’s surface area and impairs effective gas exchange. Percolation theory, which examines the flow of fluids through interconnected pathways, can be used to model these structural changes and evaluate their potential effects at various levels of scale [13].

A percolation model known as a random resistor network can be used to explore how variations in elastic fibers impact lung mechanics. This model is characterized by randomly disconnected conducting bonds that increase resistance to the flow of electrical or mechanical forces [14]. It includes two interconnected units: K1, representing intact fibers, and K2, representing fragmented fibers. These units are distributed randomly within a three-dimensional lattice, with their relative proportions affecting the transmission of mechanical force throughout the lung [15].

**Figure 2:**
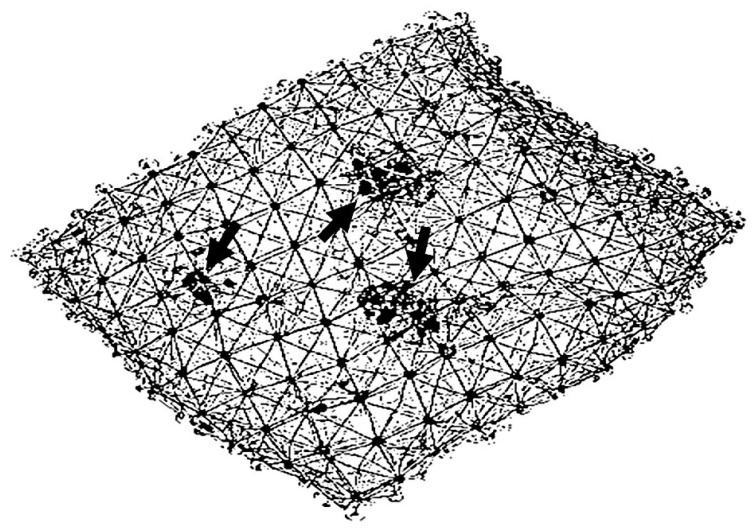
Diagram of lung elastic fiber network showing intact (solid lines) and fragmented (dotted lines) fibers. The increased mechanical strain on the remaining intact fibers facilitates their fragmentation, producing foci of alveolar wall distention and rupture (arrows) that eventually become confluent.

When the proportion of K2 units is low, forces are distributed broadly across the more robust K1 units, resulting in the minimal disruption of the lung structure. However, as the number of K2 units increases, indicating damage to the elastic fibers, forces become more concentrated in the remaining K1 units. The heightened strain on K1 units accelerates their breakdown into K2 units, leading to a loss of elastic recoil, the hyperinflation of the alveoli, and the rupture of alveolar walls (Figure 2). These structural changes are reflected at a larger scale by increased lung compliance and reduced gas exchange, which may result in respiratory failure.

### 1.2. The Relationship Between Elastic Fiber Fragmentation and Transmission of Mechanical Force

As described in a previous publication, we can utilize the formula for current density to describe the transmission of forces through the K1 and K2 units in the following manner [5]:

*j* = *ρ*_1_*v*_1_ + *ρ*_2_*v*_2_

The current flow through these bonds can be likened to the transmission of mechanical forces, with *ρ*_1_*v*_1_ and *ρ*_2_*v*_2_ representing the force density in the K1 and K2 units, respectively. Although there are inherent differences between mechanical forces and electrical currents, we can characterize the impact of structurally modified fibers in relation to the conductance parameters using the following formula:

$$G=\frac{A}{\rho l}$$

where *G* is conductance, and *A*, *l*, and *ρ* represent the conducting material’s area, length, and resistivity, respectively [5].

**Figure 3:**
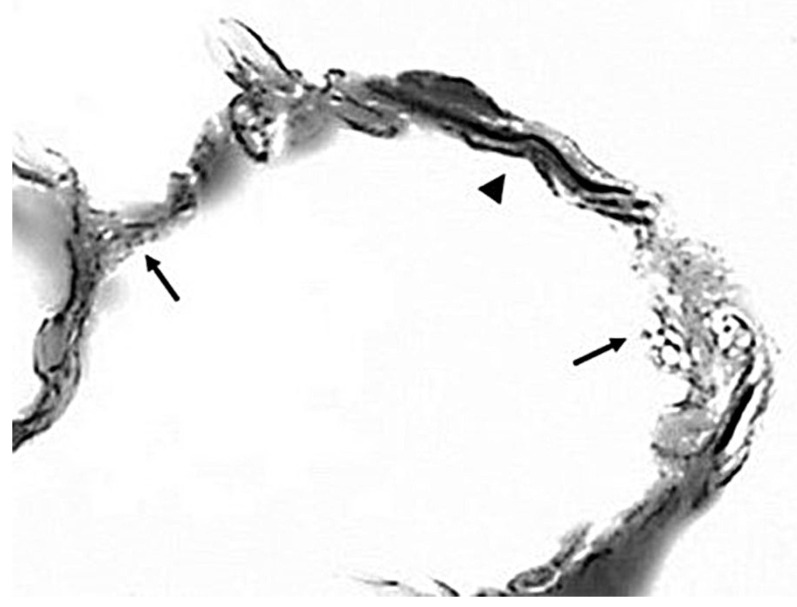
Photomicrograph of unraveled (arrowhead) and fragmented (arrows) elastic fibers following treatment of the lung with elastase and LPS. The breakdown of these fibers releases proinflammatory elastin peptides that facilitate the progression of airspace enlargement. Reprinted with permission from [16]. Orcein stain; original magnification: 1000×.

Regarding the lung, the fragmentation and stretching of the elastic fiber network lead to a reduction in surface area and an increase in length. This disruption interferes with the normal transmission of mechanical forces and increases strain on the alveolar walls (Figure 3).

### 1.3. The Proinflammatory Activity of Structurally Modified Elastic Fibers

A hamster model of pulmonary emphysema was established using elastase and LPS to examine the link between lung inflammation and the damage caused to elastic fibers [16]. To amplify the effect of LPS relative to elastase, the hamsters received a single low dose of elastase, accompanied by a shortened interval before administering LPS. This modification led to LPS having a more pronounced effect, enabling the exploration of possible synergistic interactions between the two agents. Unlike earlier studies that involved multiple weekly elastase treatments followed by LPS, this model focused on a single dose and a compressed timeline between the two substances [17,18].

The model’s objective was to assess whether pretreatment with elastase modified the structure of elastic fibers, making them more susceptible to subsequent damage from LPS. The results indicated that the combined treatment of elastase and LPS significantly increased both the total leukocyte count and the percentage of neutrophils in bronchoalveolar lavage fluid (BALF) when compared to the groups that received either elastase with saline, saline with LPS, or saline alone.

The proinflammatory effects of elastin peptides released from degraded elastic fibers were also investigated in the LPS model of lung injury [16]. The simultaneous intratracheal administration of elastin peptides and LPS led to a significant increase in neutrophil levels and DID in BALF compared to treatments with either substance alone.

In vitro experiments were conducted using BALF macrophages from untreated animals to further explore the chemotactic properties of elastin peptides in vitro [16]. When administered separately, elastin peptides and LPS significantly enhanced macrophage chemotaxis relative to the control group. However, combining the two agents resulted in an even greater increase in chemotaxis.

**Figure 4:**
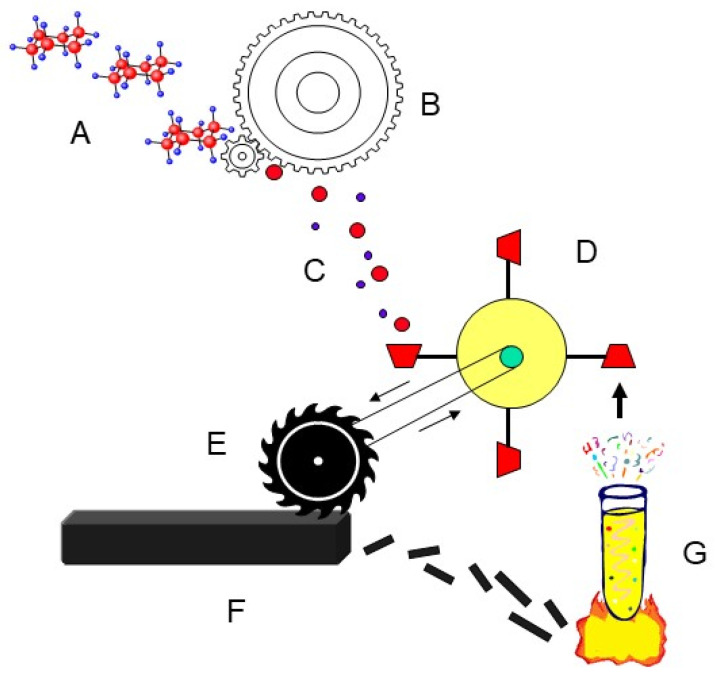
A graphic representation of the self-perpetuating inflammatory process involving the release of elastin peptides from damaged elastic fibers. Tobacco smoke and other oxidants (A) enter biochemical machinery (B) and are broken down into free radicals (C) which turn on inflammatory engine (D) that activates elastases (E) which break down elastic fibers (F), fueling release of cytokines (G) that speed up inflammatory engine.

These findings support the concept that elastases play multiple roles in the pathogenesis of pulmonary emphysema. The degradation of elastic fibers causes the release of elastin peptides, resulting in a self-perpetuating mechanism involving the release of cytokines, the recruitment of inflammatory cells, and an increase in elastic fiber degradation (Figure 4).

### 1.4. DID as a Biomarker of Elastic Fiber Injury

Given the low turnover rate of elastic fibers in healthy lungs, the DID levels in body fluids may serve as a biomarker for elastin degradation in pulmonary emphysema. Studies have shown that individuals with this condition exhibit elevated blood and urine crosslink levels [6,7,8]. Higher plasma DID levels have also been associated with a decreased lung mass, as measured by high-resolution CT imaging [8]. These findings suggest that measuring DID levels could be a practical approach for monitoring the progression of airspace enlargement in pulmonary emphysema.

Our laboratory investigated the peptide-free DID levels in hamsters’ lungs subjected to cigarette smoke and LPS to model pulmonary emphysema [9]. The findings revealed a positive correlation between the free DID levels and the enlargement of the alveolar diameter, supporting the potential of these crosslinks as biomarkers for airspace distention. A similar pattern was noted in postmortem human lung tissues, where free lung DID levels markedly increased when the alveolar diameter exceeded 400 µm [19]. This accelerated release of free DID from damaged elastic fibers may presage the appearance of clinically evident disease.

The analysis of total DID in lung tissue sections highlighted a phase transition marked by a rapid increase in the crosslink density when the alveolar diameter surpassed 300 µm and a stabilization of the process at 400 µm [19]. This finding suggests that the initial stages of airspace expansion are characterized by a dynamic balance between the injury and repair of elastic fibers, with an increased crosslink density associated with enhanced elastin deposition. However, as time progresses, this repair mechanism may become impaired, resulting in a scenario where fiber degradation outpaces their resynthesis. This decline reduces structurally intact elastic fibers, culminating in a phase transition involving the rupture of alveolar walls and a progression to a more severe disease state, which is less amenable to treatment.

### 1.5. Measurement of DID Using Mass Spectrometry

Mass spectrometry operates on the principle of ionizing chemical species and sorting the ions based on their mass-to-charge ratio (*m*/*z*). The process involves three main steps: ionization, mass analysis, and detection. Regarding the measurement of DID, the sample needs to be prepared and ionized by exposure to a high-energy electromagnetic field. The generated ions are then analyzed to determine their mass and abundance, allowing for the identification and quantification of DID. The use of liquid chromatography in combination with tandem mass spectrometry (LC-MS/MS) has significantly improved the measurement of these crosslinks, which are now detectable at nanogram levels [5,6,7,8,9].

Nevertheless, the acceptance of DID as a biomarker of alveolar wall injury will require the standardization of the methods used to measure their levels in body fluids [20]. Biological samples are inherently complex, containing numerous molecules that can co-exist with DID. The presence of proteins, peptides, lipids, and metabolites can interfere with the detection of DID by mass spectrometry [21]. Matrix effects can lead to variable ionization efficiencies, resulting in unreliable quantification. The process of extracting DID from biological samples can significantly influence measurement outcomes. Variability in the sample preparation techniques (e.g., the use of different extraction solvents, purification methods, and sample storage conditions) can introduce inconsistencies that complicate the standardization process.

These issues may be compounded by differences in the sensitivity, resolution, and analytical design of the mass spectrometer (e.g., electrospray ionization vs. matrix-assisted laser desorption/ionization). Developing a consensus method and ensuring adherence to standardized protocols is crucial for the comparability of data in multicenter studies. This process necessitates rigorous method validation, including specificity, sensitivity, accuracy, precision, and reproducibility.

### 1.6. Clinical Implementation of DID Measurements

An important application of this biomarker involves assessing the effectiveness of potential therapeutic agents for pulmonary emphysema. Both free and total urine DID have been utilized for this purpose, but neither parameter is specific for lung elastic fiber injury. In contrast, the total DID levels in sputum, which have also been evaluated in clinical trials, may serve as a more sensitive and specific indicator of COPD progression, but the difficulty of obtaining adequate amounts of this fluid from the lungs of COPD patients may adversely affect the reproducibility of this approach.

These issues indicate that measuring the free DID levels in bronchoalveolar lavage fluid (BALF), breath condensate, and lung biopsies may offer more reliable alternatives to plasma, urine, or sputum. The high sensitivity of LC-MS/MS may allow the free and total DID to be quantified in minute fragments of fresh and formalin-fixed lung tissue.

**Figure 5:**
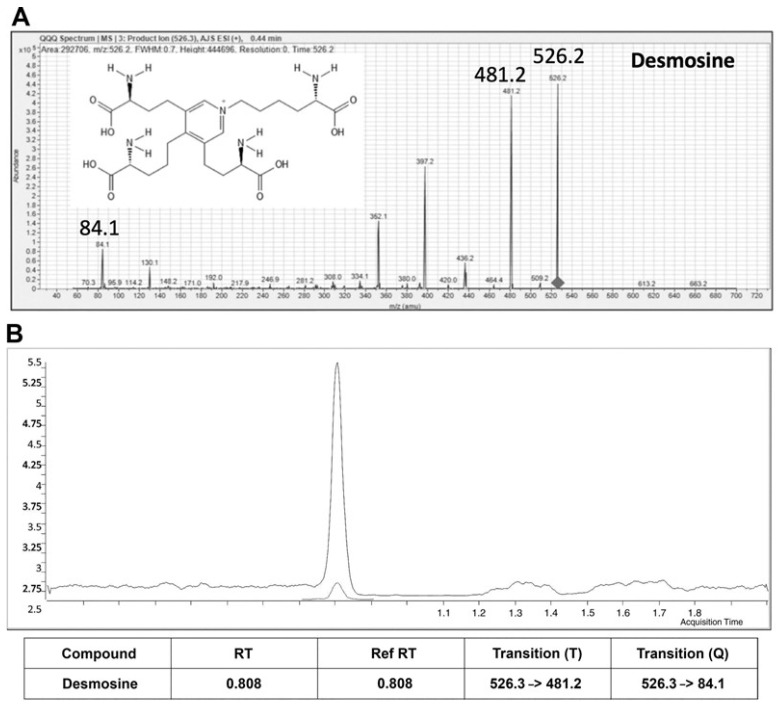
(**A**) Mass spectrometry profile showing the transitional ions used to identify desmosine. These ions result from the exposure of the chromatographically separated DID to a high-energy electromagnetic field. (**B**) The large chromatographic peak consists of DID derived from formalin-fixed tissue sections. The smaller coeluting peak is a deuterium-labeled DID standard used to identify the composition of the main peak. Reprinted with permission from [19].

Our laboratory has previously demonstrated the feasibility of measuring DID in slide sections from postmortem lungs (Figure 5) [19]. While these histological sections are much larger than the ones derived from bronchoscopy or CT-guided needle aspiration, using multiple biopsy slides for DID measurements might compensate for this difference. However, the risk of lung complications associated with these invasive procedures, particularly atelectasis following needle biopsies, may limit their use as a diagnostic tool.

The observation that the formalin fixation of lung specimens did not significantly alter the measurement of free DID indicates that the crosslinks may attach to other molecules, hindering their removal from the tissue [9,19]. One study demonstrated that DID can bind to fatty acids through electrostatic interactions with positively charged quaternary ammonium groups [21]. These attachments could then be disrupted during the chromatographic phase of DID analysis, facilitating the measurement of these crosslinks.

Although further research is required to validate free DID as an indicator of alveolar wall injury in pulmonary emphysema, its correlation with airspace enlargement could enable its use as a surrogate endpoint in clinical trials. Additionally, elucidating the relationship between localized alveolar wall alterations and the loss of elastin crosslinks in postmortem lung specimens may shed light on the patterns of disease progression in pulmonary emphysema, ultimately enhancing the diagnosis and treatment of the condition.

### 1.7. Mass Spectrometry vs. Other Assay Procedures

The main alternative to mass spectrometry for measuring DID is immunoassays that utilize antibody–antigen interactions, including the enzyme-linked immunosorbent assay (ELISA), radioimmunoassay (RIA), and lateral flow tests [20]. Immunoassays require less technical expertise and are designed to have quick turnaround times, allowing for faster decision-making in clinical settings. They are also less expensive than mass spectrometry and can be performed in various laboratory settings without specialized equipment. However, immunoassays may suffer from cross-reactivity, where antibodies bind to unintended targets, leading to false positives or negatives. While many immunoassays are sensitive, they may not reach the detection limits that mass spectrometry can achieve.

Both mass spectrometry and immunoassays have unique strengths and weaknesses concerning diagnostic accuracy [20]. Mass spectrometry is often favored for its high sensitivity, specificity, and ability to analyze complex mixtures, whereas immunoassays are valued for their simplicity, speed, and cost-effectiveness. The choice between these methods often depends on the specific application, the required analytical parameters, and logistical considerations in clinical or research settings. In the case of DID measurement, the need for extreme accuracy, reproducibility, and high sensitivity favors the use of mass spectrometry.

### 1.8. Alternative Biomarkers for Diagnosis and Monitoring Pulmonary Emphysema

Additional studies have focused on the proteomic analysis of bronchial lavage fluid, plasma, and lung tissue to identify proteins associated with emphysema. A deficiency of alpha-1 antiprotease has long been recognized as a genetic risk factor for pulmonary emphysema [22]. Genetic screening for AAT deficiency can help identify individuals at a high risk of developing this disease at an early age.

Another potential biomarker is fibrinogen, an acute-phase protein produced by the liver and an essential component of the coagulation cascade. In the context of pulmonary emphysema, research has focused on fibrinogen as a potential biomarker. Fibrinogen levels correlate with the severity of emphysematous changes and are also elevated in response to inflammation associated with COPD exacerbations [23,24,25]. However, increases in this biomarker are also seen in conditions that may not be directly related to COPD, limiting its use as an isolated diagnostic tool [24].

Surfactant Protein D (SP-D), a component of the pulmonary surfactant system involved in the immune response within the lungs, has also been studied as a potential biomarker of pulmonary emphysema [26]. It is crucial in maintaining lung homeostasis, regulating inflammation, and protecting against respiratory pathogens. Elevated SP-D levels have been associated with increased alveolar damage in pulmonary emphysema but may have limited use as a biomarker due to their lack of specificity for this disease.

Regarding genetic biomarkers, recent genome-wide association studies have identified several single-nucleotide polymorphisms (SNPs) associated with pulmonary emphysema, and variants in the SERPINA1 gene, HHIP tissue remodeling genes and surfactant protein genes have been linked to an increased risk of the disease [27,28]. However, this class of biomarkers does not play a role in diagnosing lung abnormalities or determining therapeutic efficacy.

## 2. DID as a Biomarker for Combined Pulmonary Fibrosis and Emphysema

### 2.1. Combined Pulmonary Fibrosis and Emphysema

The proximity of enlarged airspaces and thickened alveolar walls in some instances of COPD has led to the emergence of a new classification called combined pulmonary fibrosis and emphysema (CPFE) [29,30,31]. While the precise mechanisms underlying CPFE remain unclear, they may be related to the effects of cigarette smoke, which can cause both airspace dilation and interstitial fibrosis. The combination of smoke exposure and structural alterations in elastic fibers may intensify the subacute inflammatory response associated with pulmonary emphysema, resulting in fibrotic lesions overlaying already distended and ruptured alveolar walls. Consequently, the coexistence of pulmonary emphysema and interstitial fibrosis may reflect interconnected events rather than distinct disease processes.

This hypothesis is supported by a study involving α1-antiproteinase-deficient mice treated with bleomycin (BLM), which showed that the enlargement of airspaces due to emphysema occurs before the onset of interstitial fibrosis [32]. Both conditions are linked to elevated neutrophil elastase activity, and using an inhibitor for this enzyme significantly reduces the severity of both disease processes. These results indicate that neutrophil elastase may play a critical role in the development of both pulmonary emphysema and pulmonary fibrosis, contributing to the degradation and subsequent remodeling of the extracellular matrix. Elastase may also be involved in the production of cytokines and their corresponding receptors that regulate the recruitment of inflammatory cells, epithelial–mesenchymal transition, and other cellular activities [33].

The combined presence of pulmonary emphysema and interstitial fibrosis may be linked to secondary pulmonary injury. Our laboratory investigated the effects of superimposed lung inflammation induced by LPS in an animal model involving short-term cigarette smoke exposure [9]. The findings revealed that the inflammatory responses in these models interact synergistically with the secondary injury, resulting in a significant worsening of lung disease. Consequently, secondary injuries, such as respiratory infections that trigger an acute exacerbation of chronic obstructive pulmonary disease (AECOPD), may play a crucial role in the development of CPFE, particularly in patients with a long-term history of smoking.

### 2.2. The Ratio of Free to Peptide Bound DID in the Transition to CPFE

Previous studies have examined elastic fiber damage in experimental models of pulmonary fibrosis and emphysema induced by the intratracheal administration of BLM and elastase, respectively [10,34]. Both forms of lung injury showed increases in BALF DID, but there were notable differences in the ratio of free to total crosslinks. In the elastase model, this ratio was significantly elevated compared to controls. In contrast, the BLM model had a much lower proportion of free DID, indicating different patterns of alveolar wall injury. The decrease in peptide-free DID observed in pulmonary fibrosis may be due to the increased deposition of extracellular matrix, which may provide structural support for elastic fibers, preventing the release of free DID. The matrix accumulation may protect the fibers against elastases and other damaging substances.

## 3. The Role of DID as a Biomarker in Clinical Trials

### 3.1. DID as a Real-Time Measure of Therapeutic Efficacy

The development of novel drugs for treating pulmonary emphysema would benefit from a real-time evaluation of their effectiveness. Clinical trials rely on pulmonary function studies as endpoints, which can take considerable time to reveal a significant therapeutic effect [35]. While high-resolution computed tomography offers a more sensitive approach to determining a successful outcome, this method also requires a prolonged interval to demonstrate positive results [36].

Several inflammatory mediators have been suggested as biomarkers for the progression of pulmonary emphysema [37]. However, free DID crosslinks may serve as a more reliable indicator of the disease, as they specifically reflect injury to the alveolar walls. Although co-existing conditions like atherosclerosis and osteoarthritis might affect specificity, measuring free DID could still be important in clinical trials. Differences in the crosslink levels between closely matched experimental and control groups could provide an accurate determination of therapeutic efficacy. Furthermore, utilizing sputum, bronchoalveolar lavage fluid (BALF), and breath condensate to measure free DID would enhance specificity for alveolar wall injury.

### 3.2. Measurement of DID in a Clinical Trial of Aerosolized Hyaluronan

While traditional treatments for pulmonary emphysema have primarily focused on elastase inhibitors, our laboratory has investigated the use of an aerosolized formulation of low-molecular-weight hyaluronan (HA), a long-chain polysaccharide [38,39]. Previous studies have shown that pretreatment with hyaluronidase can exacerbate airspace enlargement in an emphysema model induced by intratracheal elastase instillation [40]. Conversely, animals receiving HA demonstrated significantly less airspace enlargement than controls in models of emphysema caused by porcine pancreatic elastase, human neutrophil elastase, and cigarette smoke. This protective effect is believed to arise from HA’s ability to bind with elastic fibers, potentially acting as a physical barrier against agents that damage elastin [38]. The potential therapeutic effect of supplementing the extracellular matrix through exogenously administered HA is supported by evidence showing markedly decreased lung HA levels in patients with pulmonary emphysema due to alpha-1 antiprotease deficiency [41].

The interaction between HA and elastic fibers may involve the formation of electrostatic or hydrogen bonds with elastin or adjacent microfibrillar components [42]. HA’s self-aggregating properties could further enhance this process, which may provide the fibers with more robust protection against elastases [43]. Furthermore, the hydrophilic characteristics of these HA complexes may facilitate energy storage in elastic fibers, helping to reduce the mechanical strain on alveolar walls that contributes to airspace enlargement. This concept is supported by recent research showing that HA and other proteoglycans play a role in alleviating the uneven distribution of forces within the extracellular matrix [44].

Given that the degradation of elastic fibers could represent a critical pathway in pulmonary emphysema, HA may offer protection against various agents that can harm the alveolar walls. The gradual nature of pulmonary emphysema’s progression indicates that even a minor reduction in the rate of airspace enlargement could significantly influence the disease’s trajectory and lower the likelihood of respiratory failure.

**Figure 6:**
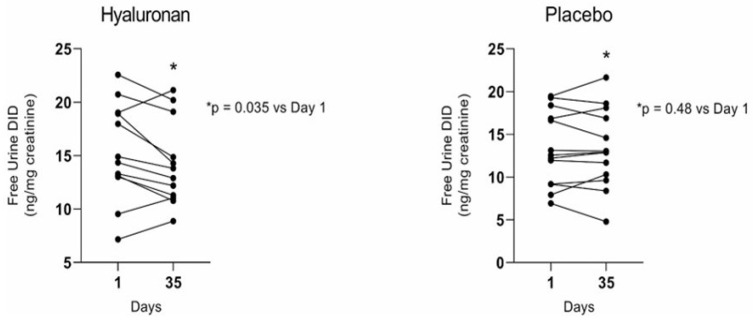
Free urine DID was significantly decreased following a 28-day clinical trial of inhaled HA in patients with alpha-1 antitrypsin deficiency-induced COPD. Measurements were made one week after the completion of treatment. No significant difference was seen in the placebo group. The findings are consistent with the experimentally demonstrated effect of HA in preventing elastic fiber degradation. The paired *t*-test was used to determine statistical significance (*p* < 0.05). Reprinted with permission from [45].

As a first step in determining the clinical efficacy of HA, a twice-daily dose of 0.1 percent HA in 3 mL of saline solution was administered over 28 days to COPD patients with alpha-1 antiprotease deficiency [39]. The plasma, urine, and sputum DID levels were measured to assess the short-term effect of treatment on elastic fiber degradation. Despite the small number of subjects and the limited time frame of the study, the inhalation of aerosolized HA significantly decreased the amount of free DID in urine. It was a more sensitive indicator of the treatment’s effect than the total DID in urine or plasma (Figure 6). While the total sputum DID may be a more sensitive indicator of therapeutic efficacy, obtaining adequate samples precluded its evaluation as an endpoint.

## 4. Therapeutic Considerations

The clinical trial examining the potential of HA to slow the progression of alveolar wall injury showed that the free DID levels in urine may act as a real-time biomarker for therapeutic efficacy [45]. While other diseases that damage elastic fibers might impact the specificity of free DID as a diagnostic tool, the biomarker could still play a crucial role in clinical studies. Significant short-term decreases in free DID would offer compelling evidence of the effectiveness of the therapeutic interventions.

While measuring free DID in BALF and breath condensate could improve the specificity of this biomarker, its acceptance as a surrogate for therapeutic efficacy will require the development of an accurate and reproducible analytic method. Liquid chromatography and mass spectrometry have become the standard method for measuring DID [46,47,48]. However, variations in the reagents and hardware used to isolate the crosslinks have led to disparate results with regard to the absolute values of DID in body fluids. Nevertheless, a recent study of the efficacy of Alvelestat©, a neutrophil elastase inhibitor, in patients with alpha-1 antiprotease deficiency-related COPD successfully demonstrated the use of blood levels of DID as a pharmacologically dynamic measure of clinical efficacy [49].

The challenge of formulating an effective treatment for pulmonary emphysema likely stems from the disease’s complex nature, where multiple interactions across various scales contribute to its progression, rather than the actions of single molecules [2,50,51,52,53]. Consequently, agents designed to inhibit specific molecular mechanisms may not effectively halt the progression of airspace enlargement, as they may overlook the structural alterations in alveolar walls resulting from these multifaceted interactions. In this context, free DID might serve as a more reliable indicator of therapeutic efficacy compared to biomarkers associated with particular inflammatory pathways.

## 5. Future Directions

While the DID biomarker is currently limited to determining the efficacy of drugs in clinical trials, developing more sensitive and specific techniques to measure these crosslinks will increase its ability to measure alveolar wall injury. Regarding mass spectrometry, a tenfold improvement in sensitivity, allowing the determination of picogram quantities of DID, may facilitate the use of breath condensate as a viable alternative to sputum, urine, and plasma. Other more abundant amino acids have been detected in breath condensate, and the abundance of free DID in the lung suggests that this medium may eventually become an accepted source of accurate and reproducible biomarker measurements. The relative volatility of DID further suggests that sufficient amounts of these amino acids can accumulate in breath condensate over a relatively short period.

A more practical and equally specific measurement of free lung DID entails using BALF DID. Although recovering the biomarker from this fluid requires more elaborate techniques, the procedure is well established and immediately applicable to the clinical setting. Regarding the acceptance of DID as a surrogate endpoint for the progression of pulmonary emphysema, the more limited designation of the biomarker as a measure of alveolar wall injury may facilitate its acceptance as an FDA-approved parameter for this disease.

The addition of this biomarker to diagnostic protocols could provide a deeper understanding of individual patient profiles and disease mechanisms, enabling tailored therapeutic strategies that address specific pathophysiological changes. The ability to monitor DID levels over time can inform treatment efficacy, significantly enhancing the precision and efficacy of personalized medical approaches.
